# Protein content of blood-derived extracellular vesicles: An approach to the pathophysiology of cerebral hemorrhage

**DOI:** 10.3389/fncel.2022.1058546

**Published:** 2023-01-19

**Authors:** Fernando Laso-García, Dolores Piniella, Mari Carmen Gómez-de Frutos, Laura Casado-Fernández, María Pérez-Mato, Elisa Alonso-López, Laura Otero-Ortega, Susana Belén Bravo, María Del Pilar Chantada-Vázquez, Lucía Trilla-Fuertes, Juan Ángel Fresno-Vara, Blanca Fuentes, Exuperio Díez-Tejedor, María Gutiérrez-Fernández, María Alonso De Leciñana

**Affiliations:** ^1^Neurological Sciences and Cerebrovascular Research Laboratory, Department of Neurology and Stroke Center, Neurology and Cerebrovascular Disease Group, Neuroscience Area La Paz Hospital Institute for Health Research—IdiPAZ (La Paz University Hospital—Universidad Autónoma de Madrid), Madrid, Spain; ^2^PhD Program in Neuroscience, Universidad Autónoma de Madrid—Instituto Cajal, Madrid, Spain; ^3^Universidad Autónoma de Madrid and IdiPAZ Health Research Institute, La Paz University Hospital, Madrid, Spain; ^4^Proteomic Unit, Health Research Institute of Santiago de Compostela (IDIS), Santiago de Compostela, Spain; ^5^Molecular Oncology and Pathology Lab, Institute of Medical and Molecular Genetics-INGEMM, La Paz University Hospital—IdiPAZ, Madrid, Spain

**Keywords:** blood, extracellular vesicles, intracerebral hemorhage, proteomics, rat

## Abstract

**Introduction:** Extracellular vesicles (EVs) participate in cell-to-cell paracrine signaling and can be biomarkers of the pathophysiological processes underlying disease. In intracerebral hemorrhage, the study of the number and molecular content of circulating EVs may help elucidate the biological mechanisms involved in damage and repair, contributing valuable information to the identification of new therapeutic targets.

**Methods:** The objective of this study was to describe the number and protein content of blood-derived EVs following an intracerebral hemorrhage (ICH). For this purpose, an experimental ICH was induced in the striatum of Sprague-Dawley rats and EVs were isolated and characterized from blood at baseline, 24 h and 28 days. The protein content in the EVs was analyzed by mass spectrometric data-dependent acquisition; protein quantification was obtained by sequential window acquisition of all theoretical mass spectra data and compared at pre-defined time points.

**Results:** Although no differences were found in the number of EVs, the proteomic study revealed that proteins related to the response to cellular damage such as deubiquitination, regulation of MAP kinase activity (UCHL1) and signal transduction (NDGR3), were up-expressed at 24 h compared to baseline; and that at 28 days, the protein expression profile was characterized by a higher content of the proteins involved in healing and repair processes such as cytoskeleton organization and response to growth factors (COR1B) and the regulation of autophagy (PI42B).

**Discussion:** The protein content of circulating EVs at different time points following an ICH may reflect evolutionary changes in the pathophysiology of the disease.

## Introduction

Stroke is the second leading cause of death and main cause of acquired disability in adults (Katan and Luft, 2018), with hemorrhagic strokes representing 10% to 25% of all stroke events (Béjot et al., 2016). Spontaneous intracerebral hemorrhage (ICH), the most common type of hemorrhagic stroke, occurs as a consequence of the acute extravasation of blood into the brain parenchyma secondary to spontaneous vascular rupture unrelated to trauma. Although ICH is associated with high mortality and morbidity and is a major cause of permanent disability (Krishnamurthi et al., 2014), there is no specific treatment with proven efficacy in improving outcomes. A better understanding of the pathophysiological mechanisms underlying the processes of damage as well as those of protection and repair triggered as a consequence of an ICH may aid in the identification of potential therapeutic targets and development of new treatments (Hemorrhagic Stroke Academia Industry (HEADS) Roundtable Participants, 2018). In this regard, the study of the content of extracellular vesicles (EVs) in serum from patients sustaining an ICH could provide important information. EVs are involved in cell-to-cell paracrine signaling (El Andaloussi et al., 2013; Qi et al., 2021) allowing for the horizontal transmission of information that relies on different molecules in the vesicle, among which proteins are of key importance (Kanada et al., 2015). EVs are secreted by most cells and their cargo is modified in response to different conditions of stress or disease (Yu et al., 2012; Yang et al., 2016). Due to these properties, EVs could be ideal biomarkers for the study of the biological processes triggered in response to the disease. Thanks to omics technologies, reliable information about their content can be obtained; specifically, proteomics, which involves the characterization of the proteome, including the expression, structure, functions, interactions, and modifications of proteins in a given sample, can provide relevant information (Domon and Aebersold, 2006; Aslam et al., 2017). We hypothesize that the analysis of blood-circulating EVs at different time points during the course of the disease can contribute key information about the pathophysiological mechanisms underlying damage and repair following an ICH that would be useful for research on new therapies. Considering that the clinical setting is not ideal for a preliminary proteomic study due to the high level of variability of clinical characteristics such as patient comorbidities and previous pathologies (Karatepe et al., 2008; O’Donnell et al., 2010), an initial approach in reproducible animal models would be more suitable to provide relevant information at the preclinical stage (Denayer et al., 2014).

For this purpose, in this study we sought to describe the number and protein content of EVs obtained from serum at different time points following the induction of an ICH in an experimental model in rats, comparing the differences with baseline.

## Materials and Methods

### Experimental procedure

**Figure 1:**
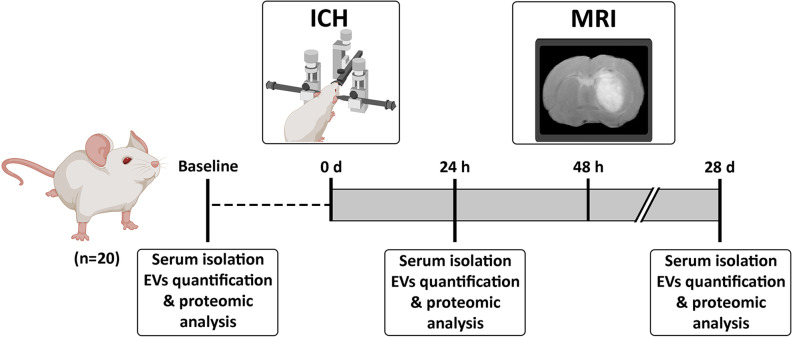
Experimental procedure. Animals were subjected to an ICH and blood-derived EVs were isolated at baseline, 24 h and 28 days post-ICH. Abbreviations: EVs, extracellular vesicles; ICH, intracerebral hemorrhage; MRI, Magnetic Resonance Imaging.

Sprague-Dawley rats (8–9 weeks old, weighing 225–275 g, male/female proportion 1:1) were used in the study (Figure 1). Anesthesia was induced using an anesthetic chamber with 8% sevoflurane in a 1 L/min oxygen flow and maintained with 4% sevoflurane in a 1 L/min oxygen flow through a facial mask. Following an intraperitoneal injection of meloxidyl (2 mg/ml; Ceva, France) to provide analgesia, the animals were placed in a stereotaxic frame. A craniotomy was performed adjacent to the bregma using the stereotaxic coordinates previously described (Otero et al., 2011) to induce an striatal hemorrhage by injection of 1 μl saline with 0.5 U collagenase type IV (Sigma-Aldrich, USA). The presence of hemorrhage was confirmed by T2-weighted spin echo magnetic resonance imaging (MRI) at 48 h post-ICH using a 7-Tesla horizontal bore magnet (Bruker Pharmascan, Germany). Blood samples were obtained from the tail vein at baseline, 24 h and 28 days after surgery. Blood tubes were centrifuged at 3,000 *g* for 15 min at 4°C and the serum was stored at −80°C until its use for the analysis of the EVs.

### Magnetic resonance imaging protocol

Images were acquired in a resolution of 7 Tesla (Bruker Pharmascan 7 Tesla, Bruker Medical Gmbh, USA) with a rapid acquisition with relaxation enhancement (RARE) sequence in axial orientations and the following parameters: number of echo images = 2 [echo time (TE): 29.54 ms and 88.61 ms], repetition time (TR) = 3,000 ms, RARE factor = 4, Av = 3, field of view (FOV) = 3.5 cm, acquisition matrix = 256 × 256 corresponding to an in-plane resolution of 137 × 137 μm^2^, slice thickness = 1.00 mm without a gap and number of slices = 16. All data were acquired using a Hewlett-Packard console running Paravision 6.0.1 software (Bruker Medical Gmbh, USA) operating on a Linux platform. Lesion volumes were visualized with Image J v1.52 (National Institutes of Health, USA).

### Extracellular vesicle isolation

EVs were isolated from serum samples using the ExoQuick ULTRA EV isolation kit (System Biosciences, Cat #EQULTRA-20A-1, USA) following manufacturer instructions as previously described (Coughlan et al., 2020). Isolated EVs were stored at −80°C until analysis.

### Extracellular vesicle characterization

EVs were characterized by: (1) Western blot; (2) transmission electron microscopy (TEM); and (3) nanoparticle tracking analysis (NTA).

Western blot was performed using specific antibodies for the detection of surface antigens in EVs: anti-CD9 (1:750, Invitrogen, Cat #MA5-31980USA), anti-CD81 (1:250, Invitrogen, Cat #MA5-13548, USA), anti-CD63 (1:1,000, Invitrogen, Cat #PA5-92370, USA), anti-Alix (1:500, Invitrogen, Cat #MA1-83977, USA) and anti-Albumin antibody (1:1,000, Invitrogen, Cat #PA5-89332 USA) as a purity control, followed by an HRP-secondary antibody (1:10,000, Abcam, UK) in a 4%–10% sodium dodecyl sulphate-polyacrylamide gel (SDS-PAGE) for electrophoresis with 20 μg of protein per lane. Band quantification was performed using a chemiluminescence method according to the manufacturer (ECL Pierce chemiluminescence; Thermo Fisher Scientific, Cat #23227, USA) in an Uvitec-Cambridge image acquisition system.

Samples were observed under the TEM (80 kV; S-3000N, Hitachi, Japan) to verify the size of the isolated EVs ranging from 30 to 200 nm. For that purpose, the EVs were fixed in 2.5% glutaraldehyde 0.1 M sodium cacodylate solution for 1 h at 4°C and postfixed with 2% osmium tetroxide for 1 h at 4°C. The pellet was dehydrated with a graded acetone series and embedded in resin, obtaining 60-nm thick sections for observation.

NTA (NanoSight LN10 instrument, Malvern Instruments, UK) was performed to determine the size and number of EVs. The sample of isolated EVs was diluted in 500 μl of sterile Phosphate Buffered Saline (PBS) for a working concentration of 1 × 10^7^–10^9^ particles/ml. Three videos of 60 s each were recorded at a shutter speed of 30.00 ms and camera level of 13. A detection threshold of three was employed for the analyses.

### Extracellular vesicle quantification

An ExoELISA-ULTRA assay kit (System Biosciences, Cat #EXEL-ULTRA-CD63-1, USA) was used for EVs quantification based on the presence of the anti-CD63 marker, following manufacturer instructions.

### Proteomic study

A mass spectrometric data-dependent acquisition (DDA) qualitative analysis and protein quantification by sequential window acquisition of all theoretical mass spectra (SWATH) data independent acquisition (DIA) was performed to determine the specific protein content of EVs at the predefined study time points. Qualitative and quantitative protein identification was performed by processing a total amount of 100 μg protein. Four microgram peptides were digested and separated using reverse phase chromatography. The gradient was created using the NanoLC 400 microflow chromatography system (SCIEX, USA) coupled to a high-speed Triple TOF 6600 mass spectrometer (SCIEX, USA) with a microflow source using a data-dependent acquisition method and 90-min gradient for qualitative analysis and 40-min gradient for library creation and grouping each EV type in a pool for SWATH quantification. For qualitative analysis or SWATH library validation, only those proteins with a false discovery rate of <1% (99% protein confidence) were selected. Once the library was prepared, the SWATH DIA method (Chantada-Vázquez et al., 2021) was created and 4 μg of each sample and its technical replicates (three samples, each run in triplicate) were analyzed in the TripleTOF 6600. The total ion chromatogram profile for each individual sample was then used in the qualitative DDA method and quantitative SWATH analysis. To confirm that the proteins obtained involved EVs content, the library was compared with the Vesiclepedia, a public reference database of EVs (Pathan et al., 2019). Proteins and the biological processes in which those proteins are involved were identified based on the UniProt database (The UniProt Consortium, 2021). For detailed explanation of the proteomic acquisition, see Supplementary Material S1. The mass spectrometry proteomics data have been deposited to the ProteomeXchange Consortium via the PRIDE (Perez-Riverol et al., 2022) partner repository with the dataset identifier PXD037246.

### Validation of the proteins of interest

A Western blot to confirm the specific proteins found in the proteomic study was performed using the same samples of EVs. Specific antibodies for the detection of UCHL1 (anti-UCHL1; 1:500, Thermo Fisher Scientific, Cat #CF504289, USA), NDGR3 (anti-NDGR3; 1:500, Thermo Fisher Scientific, Cat #PA5-101780, USA), COR1B (anti-coronin 1B; 1:500, Thermo Fisher Scientific, Cat #PA5-89926, USA), PI42B (anti-PIP4K2B; 1:500, Cell Signaling, Cat #96945, USA) and β-Actin as loading control (anti- β-Actin; 1:5,000, Sigma-Aldrich, Cat #A2228, USA) were used. The methodology for the Western blot analysis has been previously explained for extracellular vesicle characterization.

### Statistical analysis

Results were expressed as mean ± standard deviation (SD). The Kruskal–Wallis test followed by the Mann–Whitney U test were used to compare the number of EVs in each of the different study time points; *p*-values of <0.05 were considered statistically significant at a 95% confidence interval. Analyses were conducted using SPSS 23 (IBM, USA), and the figures were obtained using GraphPad Prism 8 (GraphPad software, USA).

The results from the proteomic studies were then analyzed using SWATH principal component analysis (PCA) and cluster analysis, R 3.5.3 (R Core Team, Austria) with “base,” “stats,” “gplots,” “Hmisc,” “dplyr,” and “car” packages. The PCA was applied considering the correlation matrix due to its pairwise two-sided p-values being 0 for the entire matrix, all of them therefore being statistically significant, which could account for the successful application of the PCA. The total variability of the data was 87.70% and is explained with the first two principal components. For the cluster analysis, a Student’s t-test for means comparison between samples and Euclidean distance suitable for quantitative variables and complete linkage as cluster criteria were applied. For differentially expressed protein selection, p-values were adjusted using a Benjamini-Hochberg correction, selecting proteins with a FC of >2 or <0.5 and an adjusted *p*-value of <0.05.

## Results

Twenty-nine rats were used over the course of the study. Five were not included because they did not develop an ICH. Four other rats died before completion of the observation period and were therefore excluded from the study. Animals excluded from the study were replaced by new subjects until the predefined sample size of 20 was reached.

### EVs characterization

**Figure 2:**
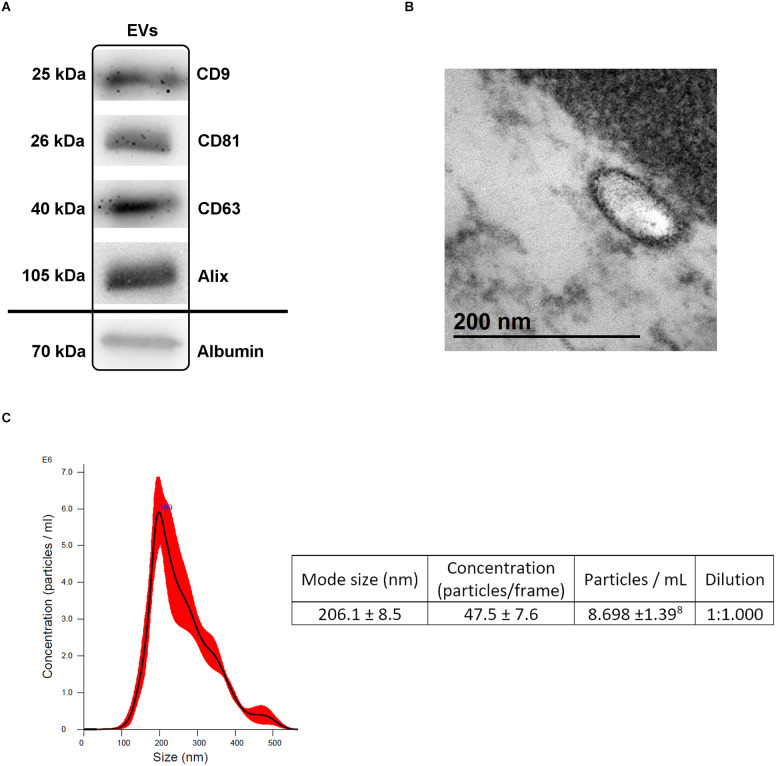
Extracellular vesicles (EVs) characterization. **(A)** Presence of specific EVs proteins (CD9, CD81, CD63, Alix) and albumin as a purity control by Western blot analysis following isolation. **(B)** Transmission electron microscopy (TEM) images illustrating the characteristic size and form of the EVs. **(C)** NTA illustrating the size and concentration of the EVs. Abbreviations: EVs, extracellular vesicles; NTA, nanoparticle tracking analysis.

Blood-derived EVs showed proteins, morphology, and size (<200 nm) specific to traditional EVs by Western blot (Figure 2A, raw data available in Supplementary Figure S1.1), TEM (Figure 2B) and NTA (Figure 2C), respectively.

### EVs quantification in serum samples

**Figure 3:**
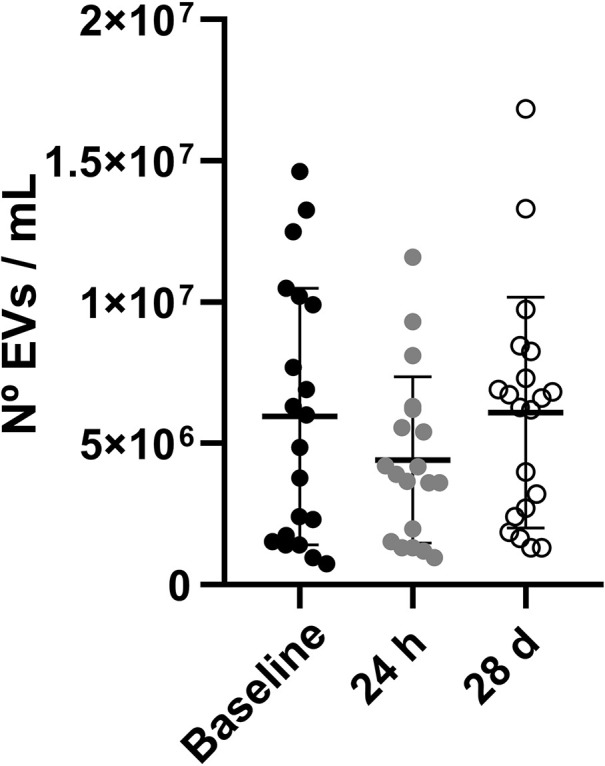
Number of blood-derived EVs at baseline, 24 h and 28 days. Data are shown as mean ± SD. Abbreviations: EVs, extracellular vesicles.

The number of blood-derived EVs at the pre-defined study time points is illustrated in Figure 3. No significant differences were found in the number of EVs at baseline (5.95 × 10^6^ ± 1.02 × 10^6^ EVs/ml), 24 h (4.42 × 10^6^ ± 6.76 × 10^5^ EVs/ml) and 28 days (6.09 × 10^6^ ± 9.12 × 10^6^ EVs/ml; *p* > 0.05).

### Proteomic analysis of EVs content

**Figure 4:**
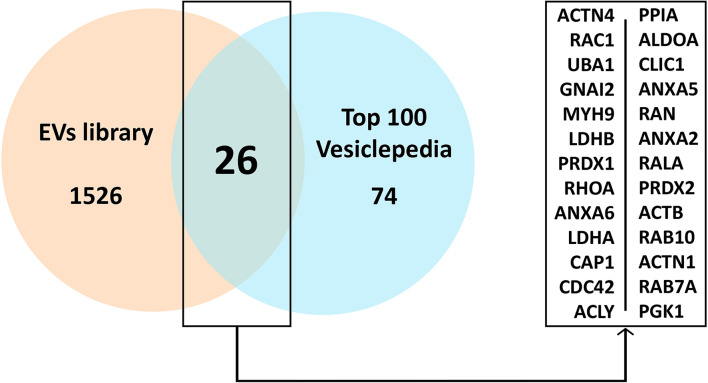
Common proteins present in the EVs study sample compared to the top 100 proteins in Vesiclepedia. Abbreviations: EVs, extracellular vesicles.

A preliminary analysis showed that our sample shared 26 proteins with the top 100 Vesiclepedia proteins (Figure 4; Pathan et al., 2019).

**Figure 5:**
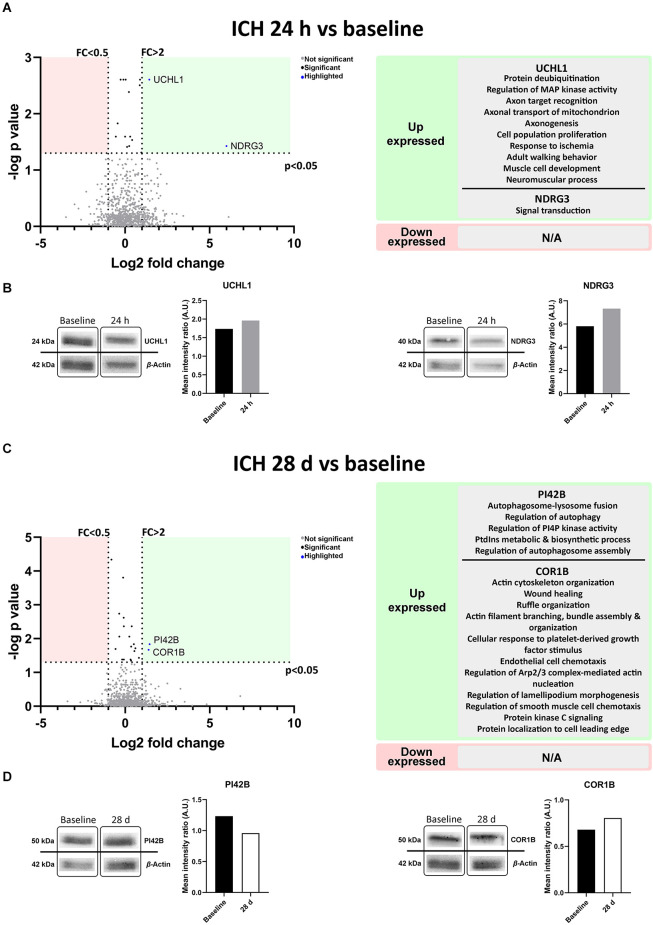
Protein content in blood-derived EVs, outlining their biological processes. **(A,C)** The volcano plots represent proteins present in the EVs compared by time point. Biological processes in which the differential proteins as described by UniProt appear to be involved are shown on the right. **(B,D)** Immunoblot assays of the selected proteins from the proteomic study. Protein detection cropped image and graphs from band quantification are shown. Abbreviations: AU, arbitrary units; EVs, extracellular vesicles; FC, fold change; ICH, intracerebral hemorrhage.

A differential expression of proteins in blood-derived EVs in the acute (24 h) and post-acute (28 days) phases of the ICH compared to baseline was observed (Figure 5A). At 24 h, ubiquitin carboxy-terminal hydrolase L1 (UCHL1), a deubiquitinating enzyme related to the ubiquitin proteasome pathway (UPP) in neurons and the mitogen-activated protein kinase (MAPK) pathway, among other biological processes, and N-Myc downstream-regulated gene 3 (NDGR3), related to signal transduction, were up-expressed vs. baseline. At 28 days, Phosphatidylinositol 5-phosphate 4-kinase type-2 beta (PI42B) related to the regulation of autophagy and lysosome fusion and Coronin-1B (COR1B) involved in the organization of the cytoskeleton, wound healing and response to growth factors were found to be up-expressed (Figure 5C). No proteins were found down-expressed compared to baseline at 24 h or 28 days.

The Western blot analysis performed to confirm the results of the proteomics, also showed higher expression of UCHL1 and NDGR3 at 24 h compared to baseline (1.96 vs. 1.74 A.U. and 7.32 vs. 5.81 A.U. respectively). At 28 days both PI42B and COR1B were tested, but only COR1B was found to be up-expressed compared to baseline (0.96 vs. 1.23 A.U. and 0.81 vs. 0.68 A.U. respectively; Figures 5B,D, raw data available in Supplementary Figure S1.2).

## Discussion

This study demonstrates that although no significant changes occur in the number of EVs; circulating EVs modify their protein content compared to baseline at different stages of evolution following an ICH. Previous works have described increased levels of EVs in autoimmune diseases and a release of EVs from different cells involved in immune responses (Tian et al., 2020; Xu et al., 2020). Considering that EVs are also secreted by almost all cell types for intercellular communication under physiological conditions (El Andaloussi et al., 2013; Kalra et al., 2016; Qi et al., 2021) and that the amount of secreted vesicles may vary depending on the status of the secreting cells, it is highly likely that only the specific EVs from cells affected in the pathological process, but not the total circulating EVs may change. We did not analyze the differential number of circulating EVs based on their cellular origin (i.e., neurons, glia or systemic immune cells), which may explain why we did not find significant changes in the total number of circulating EVs following ICH in our study. In addition, this suggests that the number of EVs in and of itself is not relevant as a biomarker for ICH. On the contrary, the differential protein cargo of these EVs in the acute and post-acute phases of an ICH compared to baseline supports the hypothesis that the protein content of circulating EVs reflects the pathophysiological mechanisms triggered as a response to the cerebral hemorrhage and thus could be used as biomarkers to better understand these processes.

Mass spectrometry allows for the identification and analysis of individual proteins in a given sample, and the detection of changes in protein levels at different stages of a disease or under different conditions (Pusch et al., 2003; Malicek et al., 2021); thus it may be used to describe the protein profile expression in EVs. To confirm the reliability of the results of our analysis, a comparison between our sample and the most common proteins located in EVs was conducted using the Vesiclepedia, a public reference database consisting of a compendium of proteins, lipids, RNA and metabolites found in EVs (Pathan et al., 2019). Based on this database, we confirmed that our study results shared 26 proteins with the top 100 Vesiclepedia proteins, supporting the assertion that the results of the proteomic analysis in fact reflected the EVs protein content.

Considering the objective to analyze the differential protein cargo in the EVs as a source of information about the pathophysiological mechanisms underlying damage and repair following an ICH, the comparison was done between the acute and post-acute phases with baseline. Proteins in the acute phase may represent mechanisms of damage, while those at later stages could be involved in mechanisms of repair.

At 24 h during the acute phase of the ICH, a differential expression of two proteins compared with baseline was observed. UCHL1 is a component of the deubiquitinating enzymes (DUBs) highly expressed in the brain, concretely in neurons, involved in the removal of ubiquitin from ubiquitinated proteins prior to entering the proteasome (Kim et al., 2003; Graham and Liu, 2017). Furthermore, UCHL1 appears to participate in the regulation of the mitogen-activated protein kinase (MAPK) pathway. MAPK is in turn involved in the regulation of a range of cellular activities including cell proliferation and migration, inflammatory responses and apoptosis by phosphorylation of various substrate proteins including transcription factors (Kim and Choi, 2010). Finally, UCHL1 can modulate the inflammatory response released by macrophages (Zhang et al., 2021) and may participate in regeneration and repair processes by enhancing cellular replacement and promoting axonogenesis as described in the Uniprot database. UCHL1 has been described as a marker of traumatic brain injury in the early post-injury period (Papa et al., 2016; Bazarian et al., 2018) and has also been found to be increased in serum from patients with ICH compared to ischemic stroke (Luger et al., 2020). Our results are in line with these data suggesting that UCHL1 detected in EVs is an early biomarker of brain damage following an ICH. However in previous works with rat models, UCHL1 was found to have increased in cerebral spinal fluid or serum following an ischemic stroke but not in hemorrhagic stroke (Ren et al., 2013). Given the biological characteristics of EVs as carriers of molecules, this discrepancy with our results can be explained because EVs can protect their content from degradation and may also contain a higher concentration of specific proteins expressed as a result of the disease. The proteomic analysis of EVs content therefore is very likely to increase the probability of finding specific biomarkers compared to the analysis in body fluids, especially in animal models in which the total amount of these biomarkers can be low.

NDGR3 was the second protein observed with up-expression in the EVs cargo during the acute phase of an ICH. This protein is a member of the NDRG family present in the nucleus of most brain cells, including neurons and Purkinje cells (Okuda et al., 2008). Under normal conditions it is degraded by the UPP, but increases in the first stages of hypoxia because its binding to lactate prevents it from being degraded by the proteasome. High levels of NDGR3 have been proposed as protection against ischemia by promoting angiogenesis and cell growth via activation of the Raf-ERK pathway in hypoxia in both *in vivo* and *in vitro* models (Yao et al., 2017). Although to our knowledge NDGR3 has not been previously described as involved in hemorrhagic stroke, the high levels of this protein in EVs suggest that it could be related to the initial cellular damage produced by the ICH.

In the post-acute phase of the ICH at 28 days, two other proteins were found to have higher expression in the EVs proteomics compared to baseline. PI42B is highly expressed in the brain and participates in the biosynthesis of phosphatidylinositol 4,5-bisphosphate (PI4, 5P2) a membrane phospholipid that is substrate of a number of signaling proteins, thus contributing to signal transduction and regulation of many cellular functions, particularly membrane dynamics (phagocytosis, endocytosis, and regulation of autophagy; The UniProt Consortium, 2021), cytoskeleton dynamics and ion channel regulation (Itoh et al., 1998; Katan and Cockcroft, 2020). The Akt signaling pathway is one of the PI4,5P2-mediated cell signaling mechanisms (Gericke et al., 2013). Activated Akt is involved in cell survival by reducing the transcription of genes involved in cell death and increasing the transcription of anti-apoptotic genes (Kim et al., 2001). COR1B was also up-expressed at 28 days. This protein has been described as involved in a wide range of biological processes potentially related to healing and restoration such as actin cytoskeleton organization, ruffle organization, endothelial chemotaxis and response to growth factors, among others (The UniProt Consortium, 2021). However, we have not found any previous works linking the biological functions of these proteins with an ICH or brain damage and, therefore, further research is needed to determine their specific role in the post-acute phase of an ICH and their potential relation to restorative processes and outcomes.

A higher expression of PI42B was not observed by immunoblotting. This may be explained by various reasons. One possible explanation is the existence of post-traslational modifications (PTMs) of the protein leading to different isoforms that were not specifically determined by the proteomic study. This protein, as can be seen in the Uniprot database has several PTMs (The UniProt Consortium, 2021). The presence of PTMs as was described by other authors (Roca-Rivada et al., 2015; The UniProt Consortium, 2021) may hinder the validation using Western blot.

In conclusion, this study reveals the differential protein content of circulating EVs in the acute and post-acute phases following an ICH compared to baseline, with a higher expression of proteins related to the response to cellular damage in the early period and of proteins related to healing and removal of debris and repair processes in later stages. These findings support the role of circulating EVs as biomarkers of the pathophysiological processes underlying ICH. Further research on the proteins described is warranted to define the molecular functions in which they are involved and their role in determining evolution and outcomes following a cerebral hemorrhage.

## Data Availability Statement

The original contributions presented in the study are publicly available. This data can be found here: https://www.ebi.ac.uk/pride/archive/projects/PXD037246.

## Ethics Statement

The experiments were carried out at the Neurological Sciences and Cerebrovascular Research Laboratory of La Paz University Hospital, Madrid, Spain. Animal care and experimental procedures were designed in accordance with the Stroke Therapy Academic Industry Roundtable (Fisher et al., 2009), RIGOR (Lapchak et al., 2013), ARRIVE (du Sert et al., 2020), and HEADS (Hemorrhagic Stroke Academia Industry (HEADS) Roundtable Participants, 2018) recommended guidelines and approved by the Ethics Committee for the Care and Use of Animals in Research (ref. PROEX 159/17) pursuant to Spanish and European Union regulations [(86/609/CEE, 2003/65/CE, 2010/63/EU) and Spanish Royal Decree (RD 1201/2005 and RD53/2013)].

## Author Contributions

Conceptualization, funding acquisition, and project administration: MG-F and MADL. Investigation and writing—original draft: FL-G, DP, and MG-F. Formal analysis and methodology: FL-G, DP, MG-F, SB, MC-V, LT-F, and JF-V. Supervision: MG-F, MADL, BF, and ED-T. Writing—review and editing: FL-G, DP, MG-F, LC-F, MP-M, EA-L, LO-O, SB, MC-V, LT-F, JF-V, BF, ED-T, MG-F, and MADL. All authors contributed to the article and approved the submitted version.
