# Supplementary material for: Protein content of blood-derived extracellular vesicles: An approach to the pathophysiology of cerebral hemorrhage
Source: Front Cell Neurosci. 2023 Jan 19;16:1058546. doi: 10.3389/fncel.2022.1058546 (PMC9912619; doi:10.3389/fncel.2022.1058546)
Supplement: Supplementary file 1 [file Data_Sheet_1.PDF]

Supplementary material (S1)

Protein identification by LC-MS/MS-DDA analysis

Protein digestion

In order to make global protein identification and quantification, an equal amount of protein (over 40 µg), from Healthy, ICH 24h and ICH 48h pooled was loaded on a 10% SDS-PAGE gel. The run was stopped as soon as the front had penetrated 3 mm into the resolving gel(1, 2) The protein band was detected by Sypro-Ruby fluorescent staining (Lonza, Switzerland), excised, and processed for in-gel, manual tryptic digestion as described elsewhere(3), gel pieces were reduced with 10 mM dithiothreitol (Sigma-Aldrich, St. Louis, MO) in 50 mM ammonium bicarbonate (Sigma-Aldrich, St. Louis, MO) and alkylated with 55 mM iodoacetamide (Sigma- Aldrich, St. Louis, MO) in 50 mM ammonium bicarbonate. Then, the gel pieces were rinsed with 50 mM ammonium bicarbonate in 50% methanol (HPLC grade, Scharlau, Barcelona, Spain), dehydrated by addition of acetonitrile (HPLC grade, Scharlau, Barcelona, Spain) and dried in a SpeedVac. Modified porcine trypsin (Promega, Madison, WI, USA) was added to the dry gel pieces at a final concentration of 20 ng/µl in 20mM ammonium bicarbonate, incubating them at 37 °C for 16 h. Peptides were extracted thrice by 20 min incubation in 40 µL of 60% acetonitrile in 0.5 % HCOOH. The resulting peptide extracts were pooled, concentrated in a SpeedVac and stored at −20 °C.

Mass spectrometric analysis (DDA acquisition)

Digested peptides (over 4 µg of each sample) were separated using Reverse Phase Chromatography. Gradient was created using a micro liquid chromatography system (Eksigent Technologies nanoLC 400, Sciex) coupled to high-speed Triple TOF 6600 mass spectrometer (Sciex) with a micro flow source. The analytical column was a silica-based reversed phase column Eksigent C18 150 × 0.30 mm, 3 mm particle size and 120 Å pore size (Eksigent, Sciex). The trap column was a YMC-TRIART C18 (YMC Technologies, Teknokroma) with a 3 mm particle size and 120 Å pore size, switched on-line with the analytical column. The loading pump delivered a solution of 0.1% formic acid in water at 10 µl/min. The micro-pump generated a flow-rate of 5 µl/min and was operated under gradient elution conditions, using 0.1 % formic acid in water as mobile phase A, and 0.1 % formic acid in acetonitrile as mobile phase B. Peptides was separated using a 90 minutes gradient ranging from 2 % to 90 % mobile phase B.

Data acquisition was performed in a TripleTOF 6600 System (Sciex, Foster City, CA) using a Data dependent workflow (DDA). Source and interface conditions was the following: ionspray voltage floating (ISVF) 5500 V, curtain gas (CUR) 25, collision energy (CE) 10 and ion source gas 1 (GS1) 25. Instrument was operated with Analyst TF 1.7.1 software (Sciex, USA). Switching criteria was set to ions greater than mass to charge

ratio (m/z) 350 and smaller than m/z 1400 with charge state of 2–5, mass tolerance 250 ppm and an abundance threshold of more than 200 counts (cps). Former target ions were excluded for 15 s. The instrument was automatically calibrated every 4 hours using as external calibrant tryptic peptides from PepCalMix.

Data Analysis

After MS/MS analysis (MS2 data), data files were processed using ProteinPilot^{TM} 5.0.1 software from Sciex which uses the algorithm Paragon^{TM} for database search and Progroup^{TM} for data grouping. Data was searched using a Rat specific Uniprot database (UniProt release 2020_05 Published on October 27, 2020. 24091 rat proteins), specifying iodoacetamide as Cys alkylation as variable modification and metionin oxidation as fixed modification. False discovery rate was performed using a non-lineal fitting method displaying only those results that reported a1% Global false discovery rate or better (4).

Protein quantification by SWATH (Sequential Window Acquisition of all Theoretical Mass Spectra)

Since its very recent technology SWATH-MS has been demonstrated to be a translational and a valuable tool in different fields being successfully applied by our group in a large variety of studies(5, 6, 15–17, 7–14)

Creation of the spectral library

In order to build the MS2 (MS/MS spectral libraries) spectral libraries, the peptide solutions were analyzed by a shotgun data-dependent acquisition (DDA) approach using micro-LC-MS/MS. To obtain a good representation of the peptides and proteins present in all samples 4 µL of each pool was separated into a micro-LC system Ekspert nLC425 (Eksigen, Dublin, CA, USA) using an Eksigent C18 150 × 0.30 mm, 3 mm particle size and 120 Å pore size (Eksigent, Sciex) at a flow rate of 5 µL/min. Water and ACN, both containing 0.1 % formic acid, was used as solvents A and B, respectively. The gradient run consisted of 5 % to 95 % B for 30 min, 5 min at 90 % B and finally 5 min at 5 % B for column equilibration, for a total run time of 40 min. As the peptides eluted, they were directly injected into a hybrid quadrupole-TOF mass spectrometer Triple TOF 6600 (Sciex, Redwood City, CA, USA) operated with a data-dependent acquisition system in positive ion mode. A Micro source (Sciex) was used for the interface between microLC and MS, with an application of 2600 V voltage. The acquisition mode consisted of a 250 ms survey (MS scan) MS1 scan from 400 to 1250 m/z followed by an (MSMS Scan) MS2 scan from 100 to 1500 m/z (25 ms acquisition time) of the top 65 precursor ions from the survey scan, for a total cycle time of 2.8 s. The fragmented precursors were then added

to a dynamic exclusion list for 15 s; any singly charged ions were excluded from the (MS/MS analysis) MS2 analysis.

The peptide and protein identifications were performed using Protein Pilot software (version 5.0.1, Sciex) with a Data was searched using a Rat specific Uniprot database (UniProt release 2020_05 Published on October 27, 2020. 24091 rat proteins), specifying iodoacetamide as Cys alkylation as variable modification and metionin oxidation as fixed modification. The false discovery rate (FDR) was set to 1 for both peptides and proteins. The MS2 spectra (MS/MS spectra) of the identified peptides were then used to generate the spectral library for SWATH peak extraction using the add-in for PeakView Software (version 2.2, Sciex) MS/MS^{ALL} with SWATH Acquisition MicroApp (version 2.0, Sciex). Peptides with a confidence score above 99% (as obtained from Protein Pilot database search) were included in the spectral library).

Relative quantification by SWATH acquisition

SWATH (Sequential Window Acquisition of all Theoretical Mass Spectra) – MS acquisition was performed on a TripleTOF® 6600 LC-MS/MS system (Sciex). Data-independent acquisition (DIA) method making 3 technical replicates for each sample were used to analyze 4 µL of peptides from each individual samples. In this case a LC-MS equipment and LC gradient described above for building the spectral library but instead using the SWATH-MS acquisition method. The method consisted of repeating a cycle that consisted of the acquisition of 100 TOF (MS/MS scans) MS2 scans (400 to 1500 m/z, high sensitivity mode, 50 ms acquisition time) of overlapping sequential precursor isolation windows of variable width (1 m/z overlap) covering the 400 to 1250 m/z mass range with a previous TOF MS1 scan (400 to 1500 m/z, 50 ms acquisition time) for each cycle. Total cycle time was 6.3 s. For each sample set, the width of the 100 variable windows was optimized according to the ion density found in the DDA runs using a SWATH variable window calculator worksheet from Sciex.

Data analysis

The targeted data extraction of the fragment ion chromatogram traces from the SWATH runs was performed by PeakView (version 2.2, Sciex) using the SWATH Acquisition MicroApp (version 2.0). This application processed the data using the spectral library created from the shotgun data. Up to ten peptides per protein and seven fragments per peptide was selected, based on signal intensity; any shared and modified peptides was excluded from the processing. Five minutes windows and 30 ppm widths were used to extract the ion chromatograms; SWATH quantization was attempted for all proteins in the ion library that was identified by ProteinPilot with an FDR below 1 %. The retention times from the peptides that were selected for each protein was realigned in each run

according to the iRT peptides corresponding a different identified proteins in each sample and eluted along the whole-time axis. The extracted ion chromatograms were then generated for each selected fragment ion; the peak areas for the protein were obtained by summing the peak areas from 10 peptides (MS1 scan) and 7 corresponding fragment ions (MS2 scan) from each peptide. PeakView computed an FDR and a score for each assigned peptide according to the chromatographic and spectra components; only peptides with an FDR below 5 % were used for protein quantization. Protein quantization was calculated by adding the peak areas of the corresponding peptides.

The integrated peak areas (processed, mrkvw files from PeakView) were directly exported to the MarkerView software (Sciex) for relative quantitative analysis. The export will generate three files containing quantitative information about individual ions, the summed intensity of different ions for a particular peptide and the summed intensity of different peptides for a particular protein. MarkerView uses processing algorithms that accurately find chromatographic and spectral peaks direct from the raw SWATH data. Data alignment by MarkerView compensates for minor variations in both mass and retention time values, ensuring that identical compounds in different samples are accurately compared to one another. For the protein/peptide library, its set of differentially expressed proteins (p-value <0.05) up regulated or down regulated proteins was selected.

SWATCH area analysis

The results from the proteomic studies were then analyzed using SWATH principal component analysis (PCA) and cluster analysis, R 3.5.3 (R Core Team, Austria) with "base," "stats," "gplots," "Hmisc," "dplyr," and "car" packages. The PCA was applied considering the correlation matrix due to its pairwise two-sided p-values being 0 for the entire matrix, all of them therefore being statistically significant, which could account for the successful application of the PCA. The total variability of the data was 87.70% and is explained with the first two principal components. For the cluster analysis, a Student's t-test for means comparison between samples and Euclidean distance suitable for quantitative variables and complete linkage as cluster criteria were applied. For differentially expressed protein selection, p-values were adjusted using a Benjamini-Hochberg correction, selecting proteins with a FC of >2 or <0.5 and an adjusted p-value of <0.05.

Bibliography for supplementary material

1. Bonzon-Kulichenko, E., Pérez-Hernández, D., Núñez, E., Martínez-Acedo, P., Navarro, P., Trevisan-Herraz, M., Del Carmen Ramos, M., Sierra, S., Martínez-Martínez, S., Ruiz-Meana, M., Miró-Casas, E., García-Dorado, D., Redondo, J. M., Burgos, J. S., and Vázquez, J. (2011) A Robust Method for Quantitative High-throughput Analysis of Proteomes by 18O Labeling. *Mol. Cell. Proteomics* 10, M110.003335

2. Perez-Hernandez, D., Gutiérrez-Vázquez, C., Jorge, I., López-Martín, S., Ursa, A., Sánchez-Madrid, F., Vázquez, J., and Yáñez-Mó, M. (2013) The intracellular interactome of tetraspanin-enriched microdomains reveals their function as sorting machineries toward exosomes. *J. Biol. Chem.* 288, 11649–61

3. Shevchenko, A., Wilm, M., Vorm, O., and Mann, M. (1996) Mass Spectrometric Sequencing of Proteins from Silver-Stained Polyacrylamide Gels. *Anal. Chem.* 68, 850–858

4. Shilov, I. V., Seymour, S. L., Patel, A. A., Loboda, A., Tang, W. H., Keating, S. P., Hunter, C. L., Nuwaysir, L. M., and Schaeffer, D. A. (2007) The Paragon Algorithm, a Next Generation Search Engine That Uses Sequence Temperature Values and Feature Probabilities to Identify Peptides from Tandem Mass Spectra. *Mol. Cell. Proteomics* 6, 1638–1655

5. Álvarez, V. J., Bravo, S. B., Chantada-Vazquez, M. P., Colón, C., De Castro, M. J., Morales, M., Vitoria, I., Tomatsu, S., Otero-Espinar, F. J., and Couce, M. L. (2021) Characterization of New Proteomic Biomarker Candidates in Mucopolysaccharidosis Type IVA. *Int. J. Mol. Sci.* 22, 226

6. Anfray, C., Mainini, F., Digifico, E., Maeda, A., Sironi, M., Erreni, M., Anselmo, A., Ummarino, A., Gandoy, S., Expósito, F., Redrado, M., Serrano, D., Calvo, A.,

Martens, M., Bravo, S., Mantovani, A., Allavena, P., and Andón, F. T. (2021) Intratumoral combination therapy with poly(I:C) and resiquimod synergistically triggers tumor-associated macrophages for effective systemic antitumoral immunity. *J. Immunother. Cancer* 9, e002408

7. Chantada-Vázquez, M. del P., García Vence, M., Serna, A., Núñez, C., and Bravo, S. B. (2021) SWATH-MS Protocols in Human Diseases. *Methods Mol. Biol.* 2259, 105–141

8. da Silva Lima, N., Fondevila, M. F., Nóvoa, E., Buqué, X., Mercado-Gómez, M., Gallet, S., González-Rellan, M. J., Fernandez, U., Loyens, A., Garcia-Vence, M., Chantada-Vazquez, M. del P., Bravo, S. B., Marañon, P., Senra, A., Escudero, A., Leiva, M., Guallar, D., Fidalgo, M., Gomes, P., Claret, M., Sabio, G., Varela-Rey, M., Delgado, T. C., Montero-Vallejo, R., Ampuero, J., López, M., Diéguez, C., Herrero, L., Serra, D., Schwaninger, M., Prevot, V., Gallego-Duran, R., Romero-Gomez, M., Iruzubieta, P., Crespo, J., Martinez-Chantar, M. L., Garcia-Monzon, C., Gonzalez-Rodriguez, A., Aspichueta, P., and Nogueiras, R. (2022) Inhibition of ATG3 ameliorates liver steatosis by increasing mitochondrial function. *J. Hepatol.* 76, 11–24

9. Gómez-Cid, L., López-Donaire, M. L., Velasco, D., Marín, V., González, M. I., Salinas, B., Cussó, L., García, Á., Bravo, S. B., Fernández-Santos, M. E., Elvira, C., Sierra, J., Arroba, E., Bañares, R., Grigorian-Shamagian, L., and Fernández-Avilés, F. (2021) Cardiac Extracellular Matrix Hydrogel Enriched with Polyethylene Glycol Presents Improved Gelation Time and Increased On-Target Site Retention of Extracellular Vesicles. *Int. J. Mol. Sci.* 22, 9226

10. Gonzalez-Rellan, M. J., Fondevila, M. F., Fernandez, U., Rodríguez, A., Varela-Rey, M., Veyrat-Durebex, C., Seoane, S., Bernardo, G., Lopitz-Otsoa, F.,

Fernández-Ramos, D., Bilbao, J., Iglesias, C., Novoa, E., Ameneiro, C., Senra, A., Beiroa, D., Cuñarro, J., DP Chantada-Vazquez, M., Garcia-Vence, M., Bravo, S. B., Da Silva Lima, N., Porteiro, B., Carneiro, C., Vidal, A., Tovar, S., Müller, T. D., Ferno, J., Guallar, D., Fidalgo, M., Sabio, G., Herzig, S., Yang, W. H., Cho, J. W., Martinez-Chantar, M. L., Perez-Fernandez, R., López, M., Dieguez, C., Mato, J. M., Millet, O., Coppari, R., Woodhoo, A., Fruhbeck, G., and Nogueiras, R. (2021) O-GlcNAcylated p53 in the liver modulates hepatic glucose production. *Nat. Commun.* 12, 1–21

11. Álvarez, J. V., Bravo, S. B., Chantada-vázquez, M. P., Barbosa-gouveia, S., Colón, C., López-suarez, O., Tomatsu, S., Otero-espinar, F. J., and Couce, M. L. (2021) Plasma Proteomic Analysis in Morquio A Disease. *Int. J. Mol. Sci.* 22, 6165

12. Otero-Ortega, L., Alonso-López, E., Pérez-Mato, M., Laso-García, F., Gómez-De Frutos, M. C., Diekhorst, L., García-Bermejo, M. L., Conde-Moreno, E., Fuentes, B., de Leciñana, M. A., Bravo, S. B., Díez-Tejedor, E., and Gutiérrez-Fernández, M. (2021) Circulating Extracellular Vesicle Proteins and MicroRNA Profiles in Subcortical and Cortical-Subcortical Ischaemic Stroke. *Biomedicines* 9, 786

13. López-López, M., Regueiro, U., Bravo, S. B., Chantada-Vázquez, M. del P., Varela-Fernández, R., Ávila-Gómez, P., Hervella, P., and Lema, I. (2021) Tear Proteomics in Keratoconus: A Quantitative SWATH-MS Analysis. *Invest. Ophthalmol. Vis. Sci.* 62, 30

14. García-Vence, M., Chantada-Vazquez, M. del P., Sosa-Fajardo, A., Agra, R., Barcia de la Iglesia, A., Otero-Glez, A., García-González, M., Cameselle-Teijeiro, J. M., Nuñez, C., Bravo, J. J., and Bravo, S. B. (2021) Protein Extraction From FFPE Kidney Tissue Samples: A Review of the Literature and Characterization of Techniques. *Front. Med.* 8, 553

15. Novelle, M. G., Bravo, S. B., Deshons, M., Iglesias, C., García-Vence, M., Annells, R., da Silva Lima, N., Nogueiras, R., Fernández-Rojo, M. A., Diéguez, C., and Romero-Picó, A. (2021) Impact of liver-specific GLUT8 silencing on fructose-induced inflammation and omega-oxidation. *iScience* 24, 102071

16. Peñas-martínez, J., Barrachina, M. N., Cuenca-zamora, E. J., Luengo-gil, G., Bravo, S. B., Caparrós-pérez, E., Teruel-montoya, R., Eliseo-blanco, J., and Vicente, V. (2021) Qualitative and Quantitative Comparison of Plasma Exosomes from Neonates and Adults. 1–21

17. Tamara, C., Nerea, L.-B., Belén, B. S., Alberto, M.-V., Aurelio, S., Iván, C., Javier, B., Felipe, C. F., and María, P. (2021) Human obese white adipose tissue sheds depot-specific extracellular vesicles and reveals candidate biomarkers for monitoring obesity and its comorbidities. *Transl. Res.* 239, 85–102

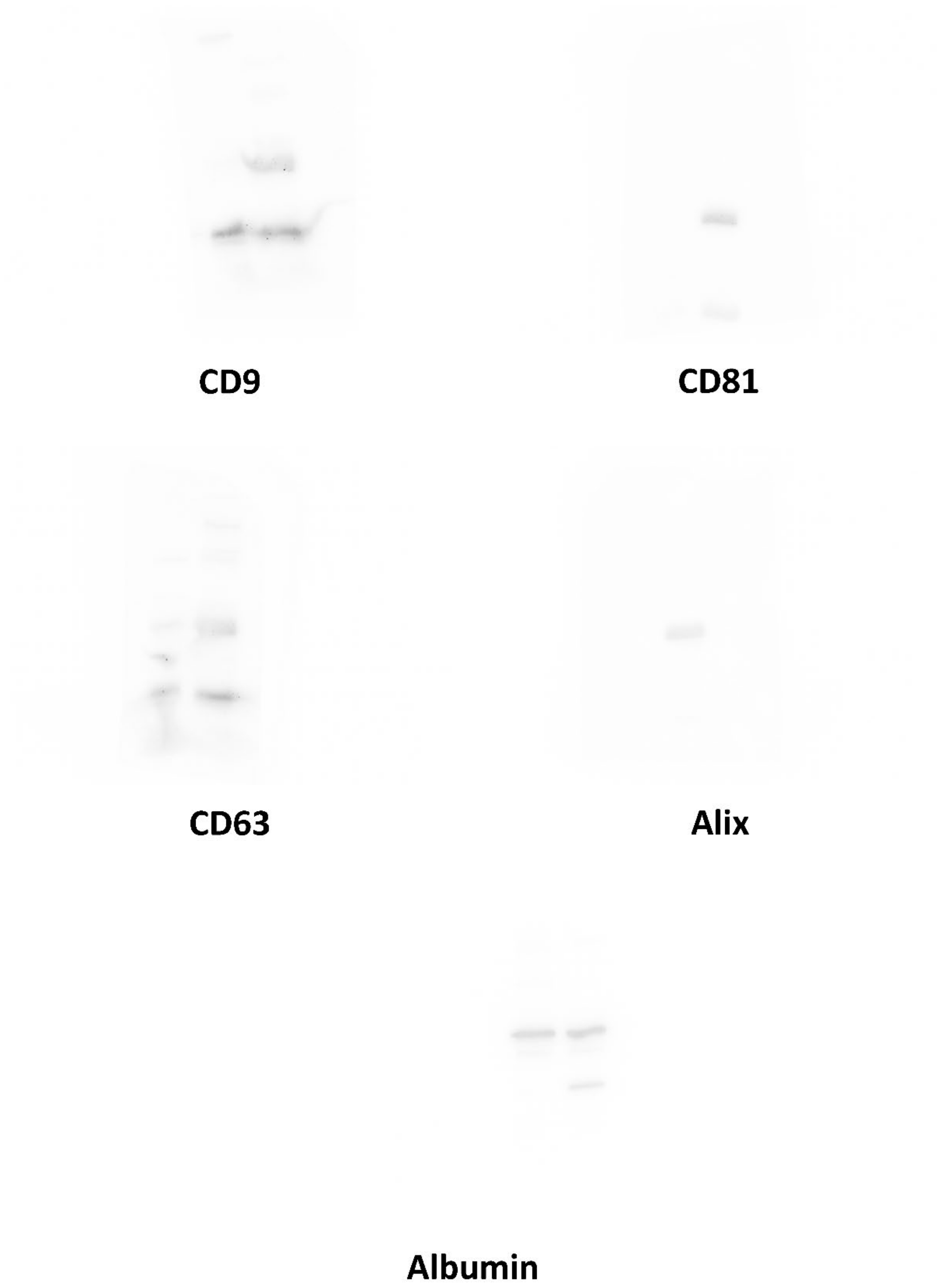

Figure S1.1. Raw images of the western blot located in figure 2A.

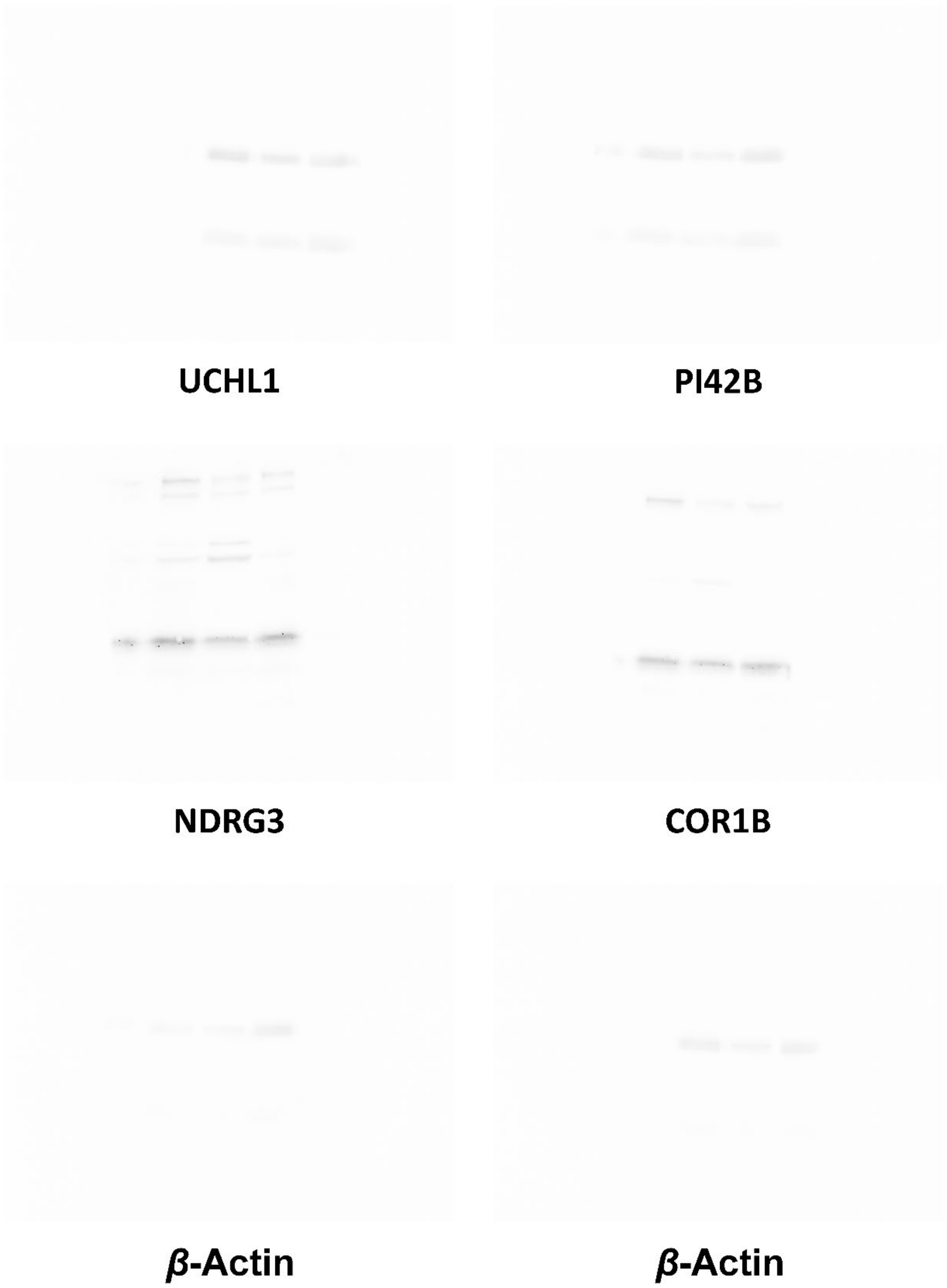

Figure S1.2. Raw images of the western blot located in figure 5B and 5D.
